# Evaluation of growth performance on family breeding of the Leizhou Black Duck: A preliminary study

**DOI:** 10.1002/vms3.263

**Published:** 2020-04-04

**Authors:** Collins Asiamah, Yuan Xue, Li‐li Lu, Kun Zou, Zhihui Zhao, Ying Su

**Affiliations:** ^1^ Department of Animal Science College of Agriculture Guangdong Ocean University Zhanjiang PR China

**Keywords:** body dimensions, body weight, correlation coefficient, growth models, slaughter performance

## Abstract

This study determined the growth performance, correlations of linear body dimensions, slaughter performance and the fitting model of two generations of Leizhou Black Ducks. Fifteen (15) male and 225 female parents forming generation 0 were selected from the Leizhou duck population. Fifteen (15) families were created in the ratio of 1 male:15 females. Eggs from all the families were collected and numbered according to the family. Generation 1 ducklings were selected and grouped into families in the same ratio. Body weights (BWs) and linear body dimensions were recorded every 2 weeks from weeks 0 to 16. Logistic, Gompertz and Von Bertalanffy models were employed to ascertain the growth model of both sexes of Leizhou Black Ducks. Except for weeks 0–2, generation 1 families had greater BWs than the generation 0 families (*p* < .05). Males from both generations had significantly higher (*p* < .01) BWs than females at 16 weeks old. Significant positive correlations were observed between BWs and measured body dimensions for both sexes except for between BW and pelvis width (PW) where negative correlations (*p* > .05) were observed for males at week 10 and females at 16 weeks old. There was a significant negative correlation (*p* < .01) between body length and PW in males at 10 weeks. The accuracy rate of Logistic, Gompertz and Von Bertalanffy models was at 0.972 and similar was obtained by all three models with Von Bertalanffy being the best model. Live weight of generation 1 before slaughter was significantly higher (*p* < .01) than generation 0, whereas no significant differences were seen in the other carcass traits. These findings provide insights into breeding Leizhou Black Duck to enhance growth performance and hybrid production.

## 1 INTRODUCTION

Duck breeding in recent times has gained much attention by breeders to select accurate animals to improve upon several traits of economic importance such as meat and egg production. A lot has been done to improve upon these traits in different duck breeds (Bhuiyan, Mostary, Ali, Hussain, & Ferdaus, 2017; Daikwo, Dike, & Dim, 2016; Ihuoma & Okata, 2016; Monleon, 2015; Ogah & Kabir, 2014; Önk, Sari, Gürcan, & Isik, 2018; Steczny, Kokoszynski, Bernacki, Wasilewski, & Saleh, 2017) except Leizhou Black Duck regardless of its reliable quality traits.

**FIGURE 1:**
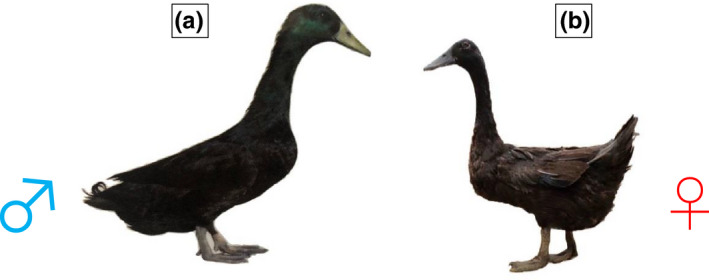
Leizhou Black Duck at 180 days old; (a) male, (b) female

Leizhou Black Duck is a duck breed widely distributed in the Leizhou Peninsula in China where there are coastal areas and tidal flats with high‐quality shallow sea biological communities such as fish, shrimps, crabs and shellfish. It has a ‘snake‐like’ head with relatively full eyes, thin neck with a long black beak and a relatively smaller but compact, long and well‐balanced body structure. Comparatively, the body size of adult female ducks is slightly smaller than the male ducks, but the body structure of the females is relatively firm and well‐proportioned and has a breeding age of 90–120 days (Figure 1). Leizhou Black Ducks have been raised on the beach for a long time and freely eat fish, shrimps, crabs and other aquatic animals. Thus, they have attained characteristics such as strong adaptability, strong disease resistance, long egg peak duration, early egg age, rich trace elements in eggs and coarse feeding tolerance (Junteng et al., 2014). As a high‐quality local duck population, genetic diversity is an excellent genetic material to improve meat and egg performance and environmental adaptability. However, because the extensive free‐range breeding of farmers is based on natural selection, the production of commercial ducks is carried out randomly. The technical content of breeding ducks is low, and there is a lack of artificial breeding system which has led to differences in individual varieties and uneven feather colour. Irregularities, alienation of genetic resources and decreased egg production are at a disadvantage in the production competition. Meanwhile, a few research has been done to identify genes involved in muscle development, functions of protein P94 and egg quality of Leizhou Black Ducks (Meng et al., 2014; Qi, 2016; Qi‐ming et al., 2013). However, to the best of our knowledge, there is no literature on the breeding of Leizhou Black Ducks on growth performance.

Growth, a change in body size per unit time, is an important characteristic in animals. Growth models such as Logistic, Gompertz, Von Bertalanffy and Richards models in different studies have been utilized to exhibit growth patterns in poultry (Narinç, Öksüz Narinç, & Aygün, 2017; Önk et al., 2018; Rizzi, Contiero, & Cassandro, 2013; Tufarelli, Selvaggi, Dario, & Laudadio, 2015; Zhao et al., 2015).

Therefore, this research focused on the breeding of two generations (0 and 1) of Leizhou Black Ducks using family breeding selection method. This was done to analyse the growth performance, the correlations between the linear body dimensions, slaughter performance and to ascertain the analytical power of the Gompertz, Logistic and Von Bertalanffy for growth estimations of the body traits in Leizhou Black Ducks.

## 2 MATERIALS AND METHODS

All the animals were maintained and studied following the National Institute of Health (NIH) guidelines for care and use of laboratory animals, and all protocols were approved in advance by the Animal Care and Ethics Committee of Guangdong Ocean University of China (No. NXY20160172).

### 2.1 Test materials

The test duck was derived from F4 selected by Guangdong Ocean University and Hengcheng Breeding Professional Cooperative in Potou District, Zhanjiang city.

### 2.2 Duck selection

**FIGURE 2:**
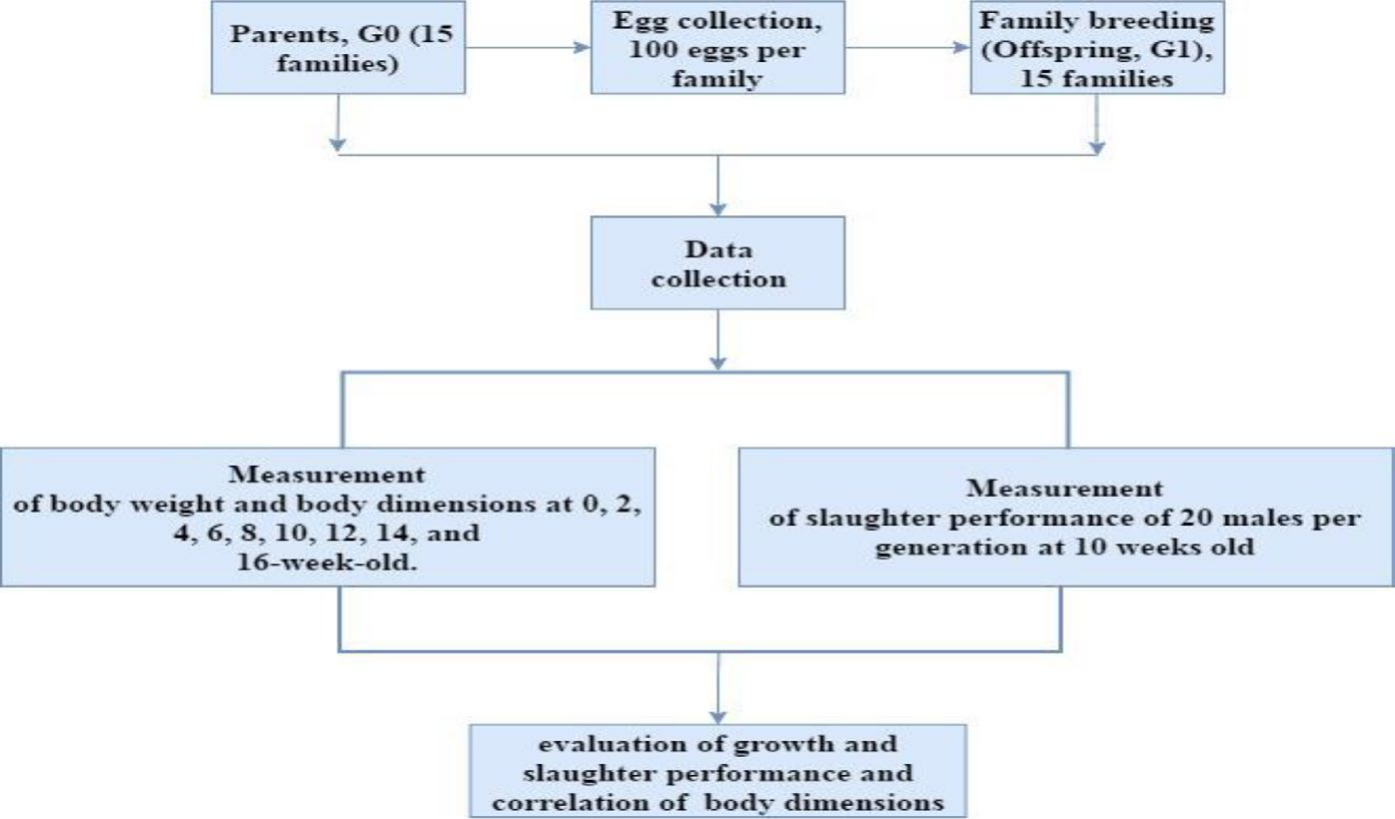
Flowchart of the experimental design

The parents used in this study comprised 15 male and 225 female Leizhou Black Ducks which were healthy for breeding. Fifteen (15) families were made from the selected animals in the ratio 1 male:15 females and placed in separate pens to form the generation 0 (G0) group. One hundred eggs were collected from each family, making 1,500 eggs in total. All the eggs were labelled according to their families. During the breeding period, 1 male and 15 female physically healthy ducks (1:15) were chosen from each family, making 15 males and 225 females. In all, 15 families were selected to form the first generation (G1) (Figure 2).

### 2.3 Housing and feeding management

From hatching to 4 weeks of age, ducklings were kept in closed buildings with windows where wood chips were used as padding and indoor raising. Heat preservation lamps as a source of heat and light were provided for more than 23 hr per day for the first 4 weeks. The temperature inside the building and rearing area (heat lamps) were kept at 24°C and 30–32°C, respectively, for the first week. The temperatures were reduced later by 1°C inside the room and 2–3°C under the heat lamps and the relative humidity was at 60%. At 4–12 weeks old, the ducklings were kept in pond tidal flats. There were duck sheds, land playgrounds and surface activities. The duck pens were naturally ventilated, and the natural light was supplemented with artificial light. There were 15 pens each with an area of 20 m^2^ containing 16 ducks each (1 male:15 females). At 13 weeks of age, eggs were ready for collection. When a duck family produced the first egg, the original basal diet was increased by 15% during egg laying. When the rate of laying reached 50%, at 17 weeks of age, it was changed to free feeding, and sufficient drinking water was supplied ad libitum throughout the rearing period. When egg laying started, the light time was gradually increased by 1 hr every week until 16 hr. The feed was obtained from Charoen Pokphand Group, Zhanjiang, Guangdong, China and the nutritional value of the diet supplied to ducks in each period is presented in Table 1.

**TABLE 1:** Nutritional value of diet at different ages of Leizhou Black Ducks

Nutritional value	Age of ducks		
	1–4 weeks	5–12 weeks	13–16 weeks
Metabolizable energy (MJ/kg)	12.1	11.4	10.4
Crude protein ≧ (%)	20.0	15.0	17.0
Crude fibre ≦ (%)	8.0	8.0	6.5
Coarse ash (%)	11.0	11.0	14.0
Calcium (%)	0.6–1.6	0.6–1.6	2.5–4.0
Total phosphorus (%)	0.3	0.3	0.4–1.5
Sodium chloride (%)	0.2–0.8	0.2–0.8	0.2–0.8
Methionine ≧ (%)	0.4	0.3	0.3

### 2.4 Data collection

The body weights (BWs) of individuals were recorded at 0, 2, 4, 6, 8, 10, 12, 14 and 16 weeks old on empty stomachs before feeding in the morning and the average BWs were calculated in grams. Measurements and calculations of the linear body dimensions were carried out following the relevant provisions of the terminologies and measurement statistics method of the agricultural industry standard of the People's Republic of China. A total of 30 Leizhou Black Ducks (half female and male) were selected randomly to measure the body traits of the first‐generation male and female ducks at 0, 2, 4, 6, 8, 10, 12, 14 and 16‐week‐old.

The body dimensions were body length (BL), length between the first cervical vertebra and the pygostyle; chest width (CW), distance between left and right glenoid cavity; chest depth (CD) was measured from the first back vertebra to the sternum; keel length (KL), distance from anterior to posterior end of the keel; pelvis width (PW), distance between two hip joints; shank length (SL) was measured from the shank joint to the extremity of the *digitus pedis*; shank girth (SG), distance around the middle part of the shank or humerus; semi‐diving length (SDL), distance from the tip of the mouth to the midpoint of the hip joint; and neck length (NL), distance between the first and last cervical vertebra. Tape measure and Vernier caliper were used to measure the body sizes in centimetres at a precision of 0.1 and 0.01 cm respectively. The data of the body sizes of the first generation were used to draw the body size growth curve of Leizhou Black Duck.

Twenty males at 10 weeks from both generations were selected for slaughter performance. The traits measured were live weight as weight of animals before slaughter, whole net carcass rate as the rate of the carcass immediately after slaughter, half net carcass rate as the rate of the carcass after removing the digestive and reproductive organs, dressing percentage measured as the rate of duck after slaughter removing all internal organs, head and inedible parts of tails and legs, breast and leg muscle rate measured as the share of muscle on the breast and leg, and sebum rate as the amount of fat on the body. All parameters for slaughter performance were weighed using an electronic balance at a precision of 0.1 g.

### 2.5 Equations of growth models

$$\text{Logistic model}:\;Y\;=\;A/\left(1+B\times e^{-kt}\right)$$

$$\text{Gompertz model}:\;Y\;=\;Ae^{-Be\left(-kt\right)}$$

$$\text{Von Bertalanffy model}:\;Y\;=\;A{\left(1-Be^{-kt}\right)}^{3}$$

where *Y* is the live weight at a particular age, *A* is the asymptotic body weight, *B* is a scale parameter, *k* is the intrinsic growth rate, *t* is the age in weeks and *e* is exponent.

### 2.6 Statistical Analysis

Test forms, field records, and data were compiled by Microsoft Excel and SPSS19.0 was used to analyse the data. Data on growth performance, BW and linear body traits and slaughter performance between the two generations were analysed using *t* test to reveal the differences. Correlation analysis among the measured body dimensions and significant tests were done using Pearson correlation coefficient method. *p*‐value < .05 was considered statistically significant.

## 3 RESULTS

### 3.1 Progress in growth performance of G0 and G1 families

**FIGURE 3:**
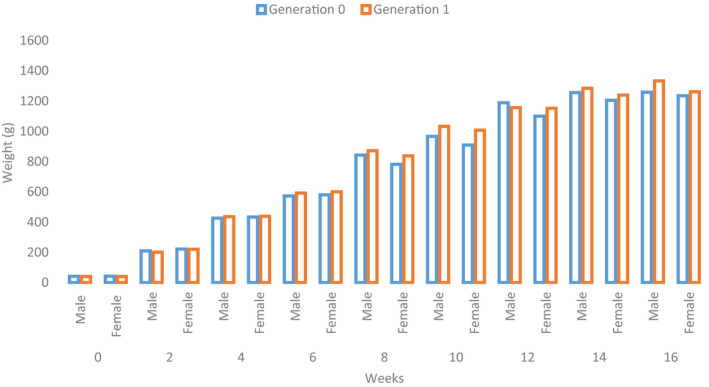
Progress of weight increase in both sexes of generations 0 and 1 families of Leizhou Black Duck

The BW of each generation increased each week of age (Figure 3). Comparatively, the BWs of G1 were higher than the G0 families except for a few weeks in which the G0 were higher than the G1, but the differences were not significant (*p* > .05) (Table 2). The weight of the first generation at 0–2 weeks was slightly lower than the zero generation. At 16 weeks old, there was a significant difference (*p* < .01) as the weight of males increased from 1258.69 g in G0 to 1333.52 g in G1 with 74.83 g increase, and the females increased from 1235.00 g in G0 to 1261.96 g in G1 with a significant increase (*p* < .01) of 26.96 g (Table 2). Except for week 0 of G1, the BWs of females in both G0 and G1 at 0–6 weeks old were greater than the males but were not significant; nonetheless, a change in trend occurred from weeks 8 to 16 where weights of males became higher than females. At week 16, the BWs of males were significantly higher (*p* < .01) than females in all generations of Leizhou Black Ducks.

**TABLE 2:** Body weight in generations 0 and 1 families of Leizhou Black Duck

Weeks	Gender	Generation 0		Generation 1	
		Weight (g)	Coefficient of variation (%)	Weight (g)	Coefficient of variation (%)
0	Male	41.90 ± 2.75^aC^	6.56	40.57 ± 3.68^aD^	9.07
	Female	42.16 ± 3.19^aC^	7.57	40.44 ± 3.10^aD^	7.67
2	Male	209.62 ± 39.17^AC^	18.67	200.91 ± 32.05^AD^	15.95
	Female	221.39 ± 43.80^Bc^	19.78	220.00 ± 35.19^Bc^	16.00
4	Male	426.35 ± 43.87^ac^	10.29	435.70 ± 47.14^ad^	10.82
	Female	433.66 ± 40.03^ac^	9.23	438.40 ± 44.62^ac^	10.18
6	Male	572.93 ± 77.00^ac^	13.44	591.94 ± 63.17^ad^	10.67
	Female	580.78 ± 103.65^aC^	17.85	600.08 ± 61.30^aD^	10.22
8	Male	842.95 ± 31.73^AC^	3.76	872.31 ± 40.68^AD^	4.66
	Female	781.73 ± 36.43^BC^	4.66	837.69 ± 45.07^BD^	5.38
10	Male	966.11 ± 34.10^AC^	3.53	1033.00 ± 52.43^AD^	5.08
	Female	909.15 ± 35.97^BC^	3.96	1007.32 ± 46.48^BD^	4.61
12	Male	1,188.64 ± 33.59^Ac^	2.83	1156.73 ± 32.58^Ac^	2.82
	Female	1100.33 ± 36.29^BC^	3.30	1152.13 ± 43.00^BD^	3.73
14	Male	1255.91 ± 33.54^AC^	2.67	1284.45 ± 39.50^AD^	3.08
	Female	1205.00 ± 36.35^BC^	2.77	1239.73 ± 54.39^BD^	4.39
16	Male	1258.69 ± 34.61^AC^	2.75	1333.52 ± 45.72^AD^	3.43
	Female	1235.00 ± 37.00^BC^	2.19	1261.96 ± 45.52^BD^	3.61

Notes

The comparison between different genders of the same generation is indicated by the letters a and b. The comparison between the same gender of different generations is indicated by letters c and d. The same lowercase letters in the same column indicate no significant difference (*p* > .05) and different lowercase letters indicate a significant difference (*p* < .05), and the different upper case letters indicate that the difference is significant (*p* < .01).

### 3.2 Body growth curve of Leizhou Black Duck

To comprehensively analyse the developmental characteristics of the body traits of Leizhou Black Ducks, the most commonly used three models of Logistic, Gompertz and Von Bertalanffy, which can describe the growth of poultry based on the fitting results of the curve models, were used. Table 3 shows that the three models of Logistic, Gompertz and Von Bertalanffy of the Leizhou Black Duck body traits have a good fit, above 0.972, which coincides with the actual curve. However, Von Bertalanffy had the highest correlations in most of the body traits.

**TABLE 3:** Parameters of three growth curve models for linear body traits of generation 1 male and female Leizhou Black Ducks

Traits	Model	Gender	*A*	*B*	*k*	*R* ^2^	Inflection point growth	Inflection point
Body length	Logistic	Male	22.66	2.93	0.47	.98	11.33	2.31
		Female	21.42	2.85	0.52	.97	10.71	2.00
	Gompertz	Male	22.97	1.48	0.34	.99	8.45	1.13
		Female	21.63	1.46	0.39	.98	7.96	0.95
	Von Bertalanffy	Male	23.13	0.40	0.30	.99	6.85	0.57
		Female	21.74	0.39	0.35	.98	6.44	0.47
Chest width	Logistic	Male	8.17	3.71	0.30	.99	4.09	4.40
		Female	8.91	3.38	0.24	.99	4.45	5.18
	Gompertz	Male	8.68	1.70	0.19	.99	3.19	2.74
		Female	9.89	1.64	0.14	.99	3.64	3.46
	Von Bertalanffy	Male	9.01	0.45	0.16	.99	2.67	1.84
		Female	10.58	0.44	0.11	.99	3.14	2.42
Chest depth	Logistic	Male	8.56	3.54	0.23	.97	4.28	5.62
		Female	7.87	2.92	0.22	.97	3.94	4.80
	Gompertz	Male	9.56	1.70	0.14	.98	3.52	3.83
		Female	8.55	1.51	0.14	.98	3.15	2.90
	Von Bertalanffy	Male	10.20	0.45	0.11	.98	3.02	2.80
		Female	9.01	0.41	0.12	.98	2.67	1.80
Keel length	Logistic	Male	12.70	4.74	0.25	.98	6.35	6.25
		Female	11.56	4.04	0.26	.98	5.78	5.29
	Gompertz	Male	14.12	1.98	0.15	.98	5.19	4.60
		Female	12.53	1.81	0.17	.99	4.61	3.57
	Von Bertalanffy	Male	15.18	0.51	0.12	.98	4.50	3.61
		Female	13.20	0.47	0.13	.99	3.91	2.60
Pelvis width	Logistic	Male	4.59	6.36	0.84	.99	2.29	2.19
		Female	4.46	2.39	0.56	.99	2.23	1.55
	Gompertz	Male	4.64	2.33	0.58	.99	1.71	1.46
		Female	4.63	1.26	0.42	.99	1.70	0.55
	Von Bertalanffy	Male	4.64	2.33	0.58	1.00	0.29	0.47
		Female	4.65	0.35	0.37	.99	1.38	0.09
Shank length	Logistic	Male	6.99	2.17	0.39	.99	3.50	2.00
		Female	6.74	2.18	0.41	.99	3.37	1.89
	Gompertz	Male	7.11	1.21	0.29	.99	2.62	0.65
		Female	6.84	1.21	0.31	.99	2.52	0.61
	Von Bertalanffy	Male	7.18	0.34	0.25	.99	2.13	0.02
		Female	6.89	0.34	0.27	.99	2.04	0.02
Shank girth	Logistic	Male	3.48	2.52	0.81	.99	1.74	1.14
		Female	3.50	2.42	0.70	.99	1.75	1.27
	Gompertz	Male	3.50	1.28	0.60	.99	1.29	0.41
		Female	3.52	1.24	0.52	.98	1.29	0.42
	Von Bertalanffy	Male	3.51	0.35	0.54	.99	1.04	0.08
		Female	3.53	0.34	0.46	.98	1.05	0.04
Semi‐diving length	Logistic	Male	54.64	2.79	0.39	.99	27.32	2.64
		Female	50.84	2.68	0.43	.99	25.42	2.32
	Gompertz	Male	55.73	1.44	0.28	.99	20.50	1.29
		Female	51.72	1.41	0.31	.99	19.03	1.10
	Von Bertalanffy	Male	56.33	0.39	0.25	1.00	16.69	0.64
		Female	52.19	0.38	0.28	1.00	15.46	0.51
Neck length	Logistic	Male	23.46	4.00	0.36	.97	11.73	3.89
		Female	20.37	4.00	0.44	.96	10.19	3.17
	Gompertz	Male	24.2	1.83	0.25	.98	8.90	2.40
		Female	20.78	1.83	0.32	.98	7.64	1.92
	Von Bertalanffy	Male	24.64	0.48	0.22	.98	7.30	1.66
		Female	21.02	0.48	0.27	.98	6.23	1.32

Note

*R* ^2^, correlation coefficient.

### 3.3 Correlation of body traits of males and females in the first generation

The BW of male duck was significantly positively correlated with the BL and the KL (*p* < .05) at week 4 and in female ducks, BW significantly correlated with BL, CW and KL (*p* < .05). The BL had a significant positive correlation with CW, CD, PW and SG in male ducks (*p* < .01) and with CW, CD, KL and SL in females (*p* < .05). In both male and female ducks, CW, CD, PW, SL, SG, SDL and NL significantly positively correlated with each other (Table 4). At age 10 weeks, BW significantly (*p* < .01) positively correlated with all measured body traits except PW in males and with only KL and SDL in females. The BL positively correlated (*p* < .01) with all measured body traits in both male and female ducks except with PW. The correlation between BL and PW in male ducks was significantly negative (*p* < .01). The CW, CD, SL, SG, SDL and NL had significant positive correlation (*p* < .01) with each other in both genders and PW significantly correlated with SL, SDL and NL only in females (Table 5). In male and female ducks at 16 weeks old, the BW had a significant positive correlation (*p* < .01, .05) with BL, CW, CD, KL and SDL. CD had a significant positive correlation (*p* < .01) with PW and CW in male and female ducks, respectively, whereas CW significantly had a positive correlation (*p* < .01) with SG only in female ducks (Table 6).

**TABLE 4:** The correlation coefficient among body weight and linear body traits of both male and female Leizhou Black Ducks measured at 4 weeks of age

	BW	BL	CW	CD	KL	PW	SL	SG	SDL	NL
BW	1	.391*	.386*	.310	.686**	.132	.128	.079	.014	.097
BL	.428*	1	.469*	.381*	.392*	.230	.423*	.160	.248	.120
CW	.170	.538**	1	.516*	.001	.437*	.607**	.385*	.629*	.224
CD	.308	.553**	.642**	1	.017	.409*	.655**	.324	.611**	.391*
KL	.395*	.049	.157	.162	1	.114	.211	.077	.049	.071
PW	.309	.560**	.738**	.756**	.176	1	.745*	.689**	.653**	.097
SL	.135	.212	.327	.338	.026	.355	1	.534*	.562*	.363
SG	.323	.769**	.318	.343	.195	.366*	−.018	1	.613**	.117
SDL	.223	−.035	.053	.349	.255	.102	.209	.030	1	.547**
NL	.242	.047	.092	.250	.211	.113	0.041	.191	.748**	1

Notes

The lower left corner is the correlation coefficient of the male duck, and the upper right corner is the correlation coefficient of the female duck.

Abbreviations: BL, body length; BW, body weight; CD, chest depth; CW, chest width; KL, keel length; NL, neck length; PW, pelvis width; SDL, semi‐diving length; SG, shank girth; SL, shank length.

* Significant correlation between indicators (*p* < .05).

** Significant correlation between indicators (*p* < .01).

**TABLE 5:** The correlation coefficient among body weight and linear body traits of both male and female Leizhou Black Ducks measured at 10 weeks of age

	BW	BL	CW	CD	KL	PW	SL	SG	SDL	NL
BW	1	.219	.122	.059	.736**	.157	.229	.194	.285*	.098
BL	.428**	1	.632**	.536**	.627**	.163	.556**	.641**	.774**	.605**
CW	.458**	.748**	1	.767**	.506**	.253	.699**	.604**	.623**	.545**
CD	.463**	.566**	.720**	1	.407**	.025	.595**	.560**	.430**	.448**
KL	.893**	.535**	.530**	.515**	1	.147	.485**	.569**	.598**	.479**
PW	−.187	−.343**	−.207	−.210	−.194	1	.355**	.182	.271*	.368**
SL	.453**	.460**	.515**	.679**	.450**	.020	1	.488**	.608**	.690**
SG	.355**	.600**	.688**	.544**	.449**	−.146	.412**	1	.592**	.584**
SDL	.382**	.611**	.746**	.698**	.552**	.001	.423**	.731**	1	.662**
NL	.492**	.679**	.559**	.423**	.544**	−.195	.516**	.749**	.517**	1

Notes

The lower left corner is the correlation coefficient of the male duck, and the upper right corner is the correlation coefficient of the female duck.

Abbreviations: BL, body length; BW, body weight; CD, chest depth; CW, chest width; KL, keel length; NL, neck length; PW, pelvis width; SDL, semi‐diving length; SG, shank girth; SL, shank length.

* Significant correlation between indicators (*p* < .05).

** Significant correlation between indicators (*p* < .01).

**TABLE 6:** The correlation coefficient among body weight and linear body traits of both male and female Leizhou Black Ducks measured at 16 weeks of age

	BW	BL	CW	CD	KL	PW	SL	SG	SDL	NL
BW	1	.423**	.538*	.447*	.594**	−.235	.317	.142	.065	.304
BL	.582**	1	.236	.050	−.100	−.099	.138	.005	−.125	.249
CW	.401*	.264	1	.520**	.346	−.309	.042	.477**	.098	.304
CD	.207	.184	−.274	1	.055	−.230	.021	.190	.340	.068
KL	.579**	.194	.197	.047	1	−.280	.312	.145	.020	.091
PW	.181	−.084	−.142	.536**	.049	1	−.283	.383	−.337	−.196
SL	.166	.042	0	.073	.234	−.134	1	.100	−.140	−.333
SG	.262	.095	.131	.182	.046	.249	−.132	1	.137	.147
SDL	.452*	.200	.126	.117	.108	.180	−.202	.220	1	.274
NL	.070	.155	.037	.601	.091	.141	.158	.150	−.110	1

Notes

The lower left corner is the correlation coefficient of the male duck, and the upper right corner is the correlation coefficient of the female duck.

Abbreviations: BL, body length; BW, body weight; CD, chest depth; CW, chest width; KL, keel length; NL, neck length; PW, pelvis width; SDL, semi‐diving length; SG, shank girth; SL, shank length.

* Significant correlation between indicators (*p* < .05)

** Significant correlation between indicators (*p* < .01).

### 3.4 Slaughter performance

Except mean live weights, which were significantly different between the two generations (*p* < .01), all other variables including dressing percentage, half net carcass rate, whole net carcass rate, share of leg muscle, share of breast muscle and share of sebum were not significant (*p* > .05). The mean live weight (g) of generations 0 and 1 were 960.50 ± 43.20 and 1030.80 ± 27.11 (*p* < .01) respectively (Table 7).

**TABLE 7:** Slaughter performance of male Leizhou Black Ducks of both generations

Traits	Generations	
	0	1
Live weight (g)	960.50 ± 43.20	1030.80 ± 27.11**
Dressing (%)	88.05 ± 1.38	88.29 ± 2.35
Half net carcass rate (%)	79.45 ± 1.52	78.80 ± 1.66
Whole net carcass rate (%)	70.54 ± 1.85	70.16 ± 0.86
Share of leg muscle (%)	11.25 ± 0.95	10.83 ± 0.60
Share of breast muscle (%)	9.01 ± 1.16	9.18 ± 0.69
Share of sebum (%)	16.23 ± 1.40	15.52 ± 0.79

** Significant difference between the two generations (*p* < .01).

## 4 DISCUSSION

### 4.1 Growth performance

Growth and development are two closely related processes; the latter dominates in embryo formation and the former during the postnatal stage of life (Hyánková, Novotná, Knížetová, & Horáčková, 2004). Heredity, sex, environment, nutrition and several other factors affect the growth and development processes. BW is a valuable economic trait for determining growth in animals.

No significant differences were detected in preliminary weight; however, a significant difference was observed in the final BW of male and female Leizhou Black Ducks. The BWs of Leizhou Black Duck increased with age, which was similarly reported in other duck breeds (Damaziak et al., 2014; Kim et al., 2012; Önk et al., 2018; Sari, Tilki, Önk, & Isik, 2013; Steczny et al., 2017). In this study, sex did not affect the weights of ducks in both generations at week 0. However, from the second to the sixth week, female ducks had higher BWs than their male counterparts for the two generations, which is similarly revealed in the results of a previous study (Kokoszyński et al., 2019). The trend changed from the 8th to the 16th week, where males in both generations had a significantly higher BW than females. The change and increased difference in weights of both sexes from 8th to 16th week may be due to the intensive growth of males than females at the latter stage. It is reported that the difference could be due to the effects of androgenic hormones on protein breakdown and the growth rate of skeletal muscles in males (Hong et al., 2014). It has been revealed that male ducks have higher BWs than females especially in the latter stages of growth (Kim et al., 2012; Ogah & Kabir, 2014; Önk et al., 2018; Sari et al., 2013; Steczny et al., 2017; Yakubu, 2011). The higher BW of males than females could also be because males have lower feed conversion ratio than females, thus more efficient in feed conversion (Dong & Ogle, 2003; Etuk, Abasiekong, Ojewola, & Akomas, 2006; Ihuoma & Okata, 2016). The feeding standards during this growth stage are conducive to improving the growth efficiency of Leizhou Black Ducks.

Generally, the BW performance of generation 1 ducks for both sexes at the 16th week was significantly higher than the generation 0 ducks. This could be due to the proper selection of ducklings from generation 0 family ducks for breeding. The BWs recorded in the current study was lesser than the values recorded for male and female native ducks raised under different systems (Önk et al., 2018), Aylesbury ducks (Ihuoma & Okata, 2016) and Pekin ducks (Mazurowski et al., 2015; Steczny et al., 2017). These dissimilarities could be ascribed to the differences in breed, raising systems, care and feeding.

### 4.2 Analysis of body growth curve

Modelling of growth curves is essential as it enhances the visualization of growth patterns with time, that is, the relationship between the age and BW of animals. The equations generated are useful for predicting the expected BWs and measurements of animals at a particular age (Onba & Erdem, 2011). In this study, three models (Logistic, Gompertz and Von Bertalanffy) were used to analyse the growth patterns and body measurements of Leizhou Black Ducks to identify the best model for describing the growth curves of ducks. The body measurements (BL, CW, CD, KL, PW, SL, SG, SDL and NL) increased in both males and females with increasing age as similarly reported in Pekin ducks (Onba & Erdem, 2011). Male ducks had higher measurements of the body traits than females except for the CW, which was higher in females than males. CW and depth are the essential traits that reflect the growth of animals (Saatci & Tilki, 2007).

The results showed that all three models could completely fit the overall growth of Leizhou Black Ducks, but Von Bertalanffy was the best as it gave the highest correlations in most of the body traits. However, Gompertz was seen to be the best fitting model in previous studies. Gompertz model was most fit for live weight records in chickens when compared with Logistic and Richard growth curve models (Tufarelli et al., 2015). Also, Gompertz exhibited less bias when compared to Logistic and Von Bertalanffy models in the growth of three chicken breeds (Zhao et al., 2015). Another study also concluded that the Gompertz model was more appropriate for BWs and linear body traits of male and female native ducks raised in different systems (Önk et al., 2018).

### 4.3 Correlation analysis of body traits of males and females in the first generation

In the breeding process of ducks, body size trait is an important index for evaluating the growth status of ducks. Phenotypic traits are obtained from the effects of genotype and environment, which are closely related to BW and reproduction performance. In this study, the correlation analysis between BW and other body traits for both male and female ducks in the first generation was done at ages 4, 10 and 16 weeks. At 4 weeks, the BW of male ducks was significantly positively related to the BL and KL with the maximum correlation coefficient of .428 between BW and length. The BL significantly correlated with CW, CD, PW and SG. The BW of female ducks was significantly correlated with BL, CW and KL with the maximum correlation coefficient of .686 between BW and KL. Also, there was a significant association between BL and CW, CD, KL and SL. The maximum correlation coefficient of .769 in week 4 was between BL and SG in males. At 10 weeks, the BW of male ducks was significantly and positively correlated with all measured body traits except for PW, which had a negative correlation. The maximum correlation coefficient was .893 between KL and BW in males. Also, except for PW, the BL correlated positively with all the traits measured. In the female ducks, the BW had a significant correlation with KL, whereas the BL was strongly correlated with all the traits except for the PW. The maximum correlation coefficient of .774 occurred between BL and SDL. At 16 weeks old, the BW of male ducks was positively correlated with BL, CW, KL and SDL with the maximum correlation coefficient of .582 between BW and length. In the females, a significant positive correlation occurred between BW and BL, CW, CD and KL with a maximum correlation coefficient of .594 between the weight and KL. The highest correlation in week 16 occurred between CD and NL of males with a correlation of .601.

A previous study in Muscovy duck revealed that a significant correlation was seen between the BW and the other body traits measured at 3, 5, 10, 15 and 20 weeks of age (Ogah & Kabir, 2014). Also, in the adult male Muscovy, the correlation between BWs and body traits was all significantly positive, but the females' correlations were not significant. A negative correlation was, however, identified between BW and NL and also between NL and SL (Ogah, Yakubu, Momoh, & Dim, 2011) of which similar was seen between SL and NL of females at week 16 in this study. BW was strongly correlated with body measurements for both sexes with the maximum correlation coefficient with thoracic parameter (.973 and .993) and wing length (.995 and .990) for males and females respectively (Téguia, Ngandjou, Defang, & Tchoumboue, 2008). The positive correlation among traits in this study suggests that they are influenced by the same gene. Also, the differences in correlation between traits of males and female adult ducks indicate that there are sexual variations in the genetic structure of birds (Ogah & Kabir, 2014; Yakubu, 2011).

### 4.4 Slaughter performance

According to the results, the mean live weight of ducks selected for slaughter was higher in generation 1 than generations 0. However, there were no significant differences in the other traits taken for both generations. Muscle tissue is most valued in the carcass of all animals and its weight is mainly determined by leg muscle and breast muscle weight in poultry (Górska & Mróz, 2014; Kokoszyński et al., 2015). In this study, the leg muscle values recorded were greater than the values recorded in 7 weeks old (males, 12.1% and females, 9.9%) Pekin ducks, but lower than ducks in another report (males, 13.5% and females, 13.2%) (Biesiada‐Drzazga, Charuta, Janocha, & Łeczycka, 2011; Steczny et al., 2017). The breast muscle rate recorded in this study is lower than those reported earlier in Pekin ducks (Kokoszyński & Bernacki, 2011; Steczny et al., 2017). Ducks are considered to have high body fat but the sebum rate recorded in this study for Leizhou Black Duck is lower than those recorded for Pekin ducks (Kokoszyński et al., 2015; Kowalczyk, Łukaszewicz, Adamski, & Kuźniacka, 2012; Steczny et al., 2017), which may be attributed to the differences in breed, raising systems, care and feeding.

## 5 CONCLUSION

Leizhou male ducks had better BW than females in both generations at the end of 16 weeks. Even though the females performed better than the males in the early stage, the males outperformed them in the latter stages. Comparatively, generation 1 families had higher BWs and live weights than the generation 0 families. Von Bertalanffy was the best fitting model for the overall growth of Leizhou Black Ducks. Furthermore, BWs and measured body dimensions of generation 1 males and females were usually positively correlated. This implied that BW was dependent on the other body dimensions, thus an upsurge in BW is related to the equivalent upsurge in body dimensions. These findings will serve as a basis to improve upon Leizhou Black Duck breeding and hybrid production.

## CONFLICT OF INTEREST

All authors declare no conflict of interest.

## AUTHOR CONTRIBUTION

**Collins Asiamah:** Conceptualization; Data curation; Formal analysis; Methodology; Writing‐original draft; Writing‐review & editing. **Yuan Xue:** Conceptualization; Data curation; Formal analysis; Methodology; Software. **Li‐li Lu:** Data curation; Methodology; Writing‐review & editing. **Kun Zou:** Data curation; Formal analysis; Software; Writing‐review & editing. **Ying Su:** Conceptualization; Funding acquisition; Methodology; Project administration; Supervision; Writing‐review & editing. **Zhihui Zhao:** Funding acquisition; Project administration; Supervision; Writing‐review & editing. 

## ETHICAL STATEMENT

The authors confirm that the ethical policies of the journal, as noted on the journal's author guidelines page, have been adhered to and the appropriate ethical review committee approval has been received. All the animals were maintained and studied following the National Institute of Health (NIH) guidelines for care and use of laboratory animals, and all protocols were approved in advance by the Animal Care and Ethics Committee of Guangdong Ocean University of China (No. NXY20160172).
